# Exploring the Frontiers of Cell Temperature Measurement and Thermogenesis

**DOI:** 10.1002/advs.202402135

**Published:** 2024-10-28

**Authors:** Hanliang Zhu, Haotian Xu, Yue Zhang, Jan Brodský, Imrich Gablech, Marie Korabečná, Pavel Neuzil

**Affiliations:** ^1^ School of Mechanical Engineering Northwestern Polytechnical University Xi'an Shaanxi 710072 P. R. China; ^2^ Department of Microelectronics The Faculty of Electrical Engineering and Communication Technology Brno University of Technology Technická 3058/10 Brno 616 00 Czech Republic; ^3^ Institute of Biology and Medical Genetics, First Faculty of Medicine Charles University and General University Hospital in Prague Albertov 4 Prague 128 00 Czech Republic; ^4^ Department of Laboratory Medicine Faculty of Health Care and Social Work University of Trnava in Trnava Universitne namestie 1 Trnava 918 43 Slovakia

**Keywords:** cell temperature measurement, intracellular thermogenesis, cellular thermodynamics, metabolic disorders, cancer thermotherapy

## Abstract

The precise measurement of cell temperature and an in‐depth understanding of thermogenic processes are critical in unraveling the complexities of cellular metabolism and its implications for health and disease. This review focuses on the mechanisms of local temperature generation within cells and the array of methods developed for accurate temperature assessment. The contact and noncontact techniques are introduced, including infrared thermography, fluorescence thermometry, and other innovative approaches to localized temperature measurement. The role of thermogenesis in cellular metabolism, highlighting the integral function of temperature regulation in cellular processes, environmental adaptation, and the implications of thermogenic dysregulation in diseases such as metabolic disorders and cancer are further discussed. The challenges and limitations in this field are critically analyzed while technological advancements and future directions are proposed to overcome these barriers. This review aims to provide a consolidated resource for current methodologies, stimulate discussion on the limitations and challenges, and inspire future innovations in the study of cellular thermodynamics.

## Graphical abstract

This review focuses on the mechanisms of local temperature generation within cells and the methods developed for accurate temperature assessment. Contact and noncontact techniques are introduced, including infrared thermography, fluorescence thermometry, and other innovative localized temperature measurement approaches. The mechanisms of cellular thermogenesis and the implications of their dysregulation in metabolic disorders and cancer are also discussed.

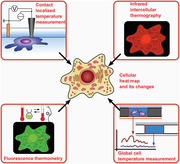

## 1 Introduction

The complex regulation of temperature within the microscopic domain of a cell^[^ ^1^ ^]^ represents a dynamic and complex aspect of cellular physiology. Cellular thermogenesis, as a part of metabolic processes involving heat production during metabolic reactions, is crucial for the proper function of cells.^[^ ^2^ ^]^ Cellular metabolism is at the heart of this thermal regulation, an inherently exothermic process where the mitochondria are essential contributors to heat generation through oxidative phosphorylation and other metabolic pathways.^[^ ^3^ ^]^ The resultant heat is a critical factor in maintaining cellular homeostasis, influencing crucial cellular components such as enzymes, membrane fluidity, and the structural integrity of nucleic acids and proteins.^[^ ^4^ ^]^ Additionally, sources of thermal energy within the cell, such as the mitochondrial electron transport chain,^[^ ^5^ ^]^ metabolic pathways,^[^ ^6^ ^]^ and muscle contractions,^[^ ^7^ ^]^ collectively contribute to maintaining a balanced thermal equilibrium. Measuring cellular temperature, a critical determinant of numerous biochemical reactions and cellular functions presents a significant challenge.^[^ ^8^ ^]^ It requires precise techniques to navigate the microscale environment and the dynamic nature of living cells.

Recent progress in thermometry and intracellular heat measurements has significantly advanced our understanding of cellular processes by enabling more precise and localized temperature assessments within living cells. Contact‐based probes^[^ ^9^ ^]^ that interact directly with cells and noncontact approaches like infrared thermography^[^ ^10^ ^]^ and fluorescence thermometry^[^ ^11^ ^]^ each offer unique insights into the subtle thermal dynamics within cells. The integration of scanning thermal microscopy with atomic force microscopy has allowed for nanoscale thermal imaging, offering unprecedented insights into the thermal dynamics of cellular structures. Innovations in fluorescence‐based thermometry, such as the development of fluorescent polymeric thermometers and fluorescent nanodiamonds, have enhanced the sensitivity and spatial resolution of temperature measurements at the subcellular level. The fluorescent dyes or probes employed for recording intracellular temperature facilitate the revelation of how cells maintain the stability of their internal environment through thermal regulatory mechanisms and the role these mechanisms play in the occurrence and progression of diseases. Furthermore, recording temperature changes in cells during drug administration can indirectly reflect the impact of drugs on cellular metabolic activities, ultimately revealing the mechanism of action of the drugs. However, technical limitations in measurement resolution, potential problems such as photobleaching in fluorescence methods,^[^ ^12^ ^]^ and the invasive nature of contact probe‐based techniques pose considerable obstacles. Furthermore, the variable nature of cellular environments and external influences necessitates careful experimental design and data interpretation. Despite these challenges, developing novel technologies and methodological approaches is breaking new ground in cellular thermodynamics. Integrating nanotechnology, refining thermosensitive dyes^[^ ^13^ ^]^ and materials,^[^ ^14^ ^]^ and applying computational models for thermal dynamics bring a new era in this field.^[^ ^13^ ^]^

This review explores the current landscape of cell temperature measurement and thermogenesis, focusing on the fundamental principles, applications, and implications in disease and therapeutics. We introduced contact and noncontact techniques, including infrared thermography, fluorescence thermometry, and other innovative approaches to localized temperature measurement. We discuss the challenges inherent in these techniques, such as the resolution limits, potential phototoxicity, and the influence of environmental factors. Additionally, we examine the technological advances that are pushing the boundaries of what is possible in the field of cellular thermodynamics. These advancements enhance our understanding of cellular functions and open new pathways for diagnosing and treating diseases.

## 2 Mechanisms of Local Temperature Generation in Cells

The production of heat in living cells depends on countless simultaneous biochemical reactions in all compartments of the cells. The cells would overheat without heat dissipation into the surrounding environment, but they are practically in a water‐based environment. Thus, this is not happening. Individual cell compartments differ in the intensity of heat production. Therefore, the existence of temperature gradients inside the cells is expected.^[^ ^14^ ^]^

**Figure 1:**
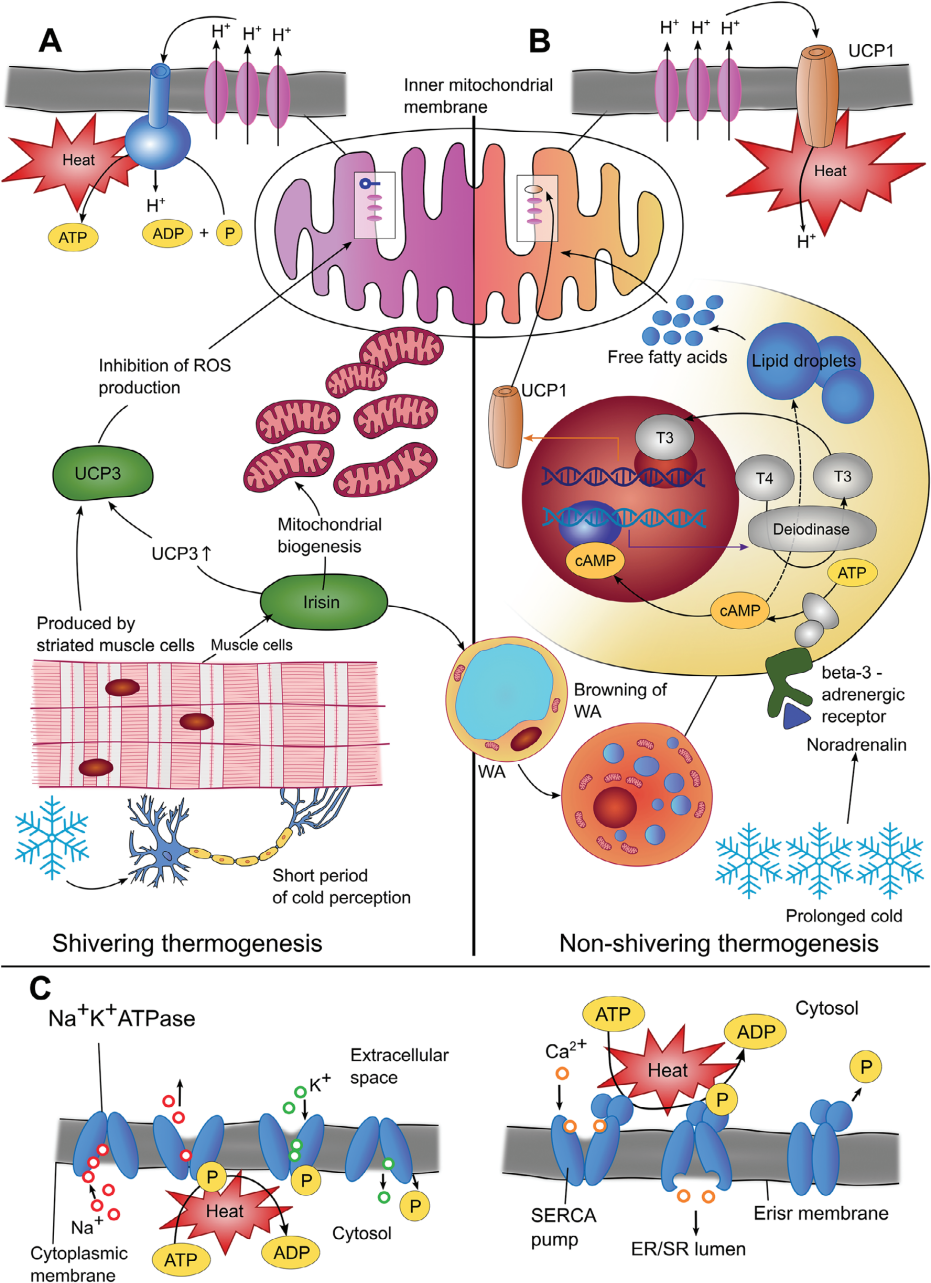
Molecular mechanisms of heat production in cells. A) Shivering thermogenesis: This process is initiated by a short period of cold perception. This results in an activity of striated muscle cells, which produce irisin and uncoupling protein 3 (UCP3). Production of irisin leads to increased mitochondrial biogenesis, increased UCP3 expression by striated muscle cells, and, eventually, the initiation of the process of browning of white adipocytes.UCP3 inhibits the production of reactive oxygen species in mitochondria, whose numbers and activity in striated muscle cells are increased.^[^ ^38^ ^]^ B) Nonshivering thermogenesis: This highly complex process occurs only in brown adipocytes activated by noradrenaline due to prolonged exposure to cold. Cyclic adenosine monophosphate (cAMP) is produced in the signaling pathway leading from the β3‐adrenergic receptor. cAMP contributes to the deiodinase II expression. This enzyme converts thyroxin (T4) to triiodothyronine (T3), which regulates the expression of uncoupling protein 1 (UCP1).^[^ ^39^ ^]^ UCP1 is the crucial molecule of non‐shivering thermogenesis, as it ensures heat production on the inner mitochondrial membrane, where it can uncouple the energy of the proton gradient from ATP production. cAMP also stimulates the production of free fatty acids (FFA) from lipids stored in droplets. FFA is the energy source in the β‐oxidation of fatty acids in the mitochondrial matrix.^[^ ^40^ ^]^ C) The activities of ion pumps localized on membranes ER/SR–endoplasmic reticulum/sarcoplasmic reticulum, WA–white adipocyte.

Mitochondria, the cellular organelles responsible for energy production, are the epicenters of thermogenesis (**Figure** **1**A,B). Oxidative phosphorylation (OXPHOS) is inherently exothermic, releasing heat as a byproduct of the electron transport chain and ATP synthesis. Fluorescence imaging techniques with fluorescent polymer probes demonstrated that the temperature in mitochondria increased by 2.4 °C and the ATP fluctuation massively decreased within 2 min during the OXPHOS process.^[^ ^15^ ^]^

In cellular thermogenesis, ion pumps, particularly the Na^+^/K^+^‐ATPase localized on the cell membrane, generate localized heat. The Na^+^/K^+^‐ATPase actively maintains cellular homeostasis by regulating ion concentrations. It hydrolyzes up to 30% of cellular ATP ^[^ ^16^ ^]^ to facilitate the active transport of Na^+^ and K^+^ against their respective concentration gradients across the plasma membrane. This localized heat release from Na^+^/K^+^‐ATPase activity is particularly significant given the abundance and constant activity of these ion pumps in almost all cell types (Figure 1C). The method based on thermoresponsive nanocomposite allows in situ monitoring of intracellular ATP variation.^[^ ^17^ ^]^

Another ion pump, Ca^2+^ ATPase, based on sarcoendoplasmic reticulum calcium ATPase (SERCA), is involved in generating heat in the endoplasmic/sarcoplasmic reticulum.^[^ ^18^ ^]^ The precise sensing of heat generation in HeLa cells exposed to calcium ions stress has been achieved using a fluorescent polymer ratiometric nanothermometer.^[^ ^19^ ^]^

The SERCA pump transports calcium ions from the cytosol back to the sarcoplasmic reticulum (SR) following muscle contraction, and this is how it plays a crucial role in cellular calcium homeostasis (Figure 1C). These pumps are highly dependent on the availability of ATP. Understanding interactions between Ca^2+^ concentration and temperature variation during the OXPHOS is crucial for exploring cell regulatory processes. A fluorescent water‐soluble polymer was developed for simultaneous intracellular temperature and Ca^2+^ gradient monitoring.^[^ ^20^ ^]^ SERCA is localized on the nuclear envelope's outer membrane, producing heat.^[^ ^21^ ^]^

**Figure 2:**
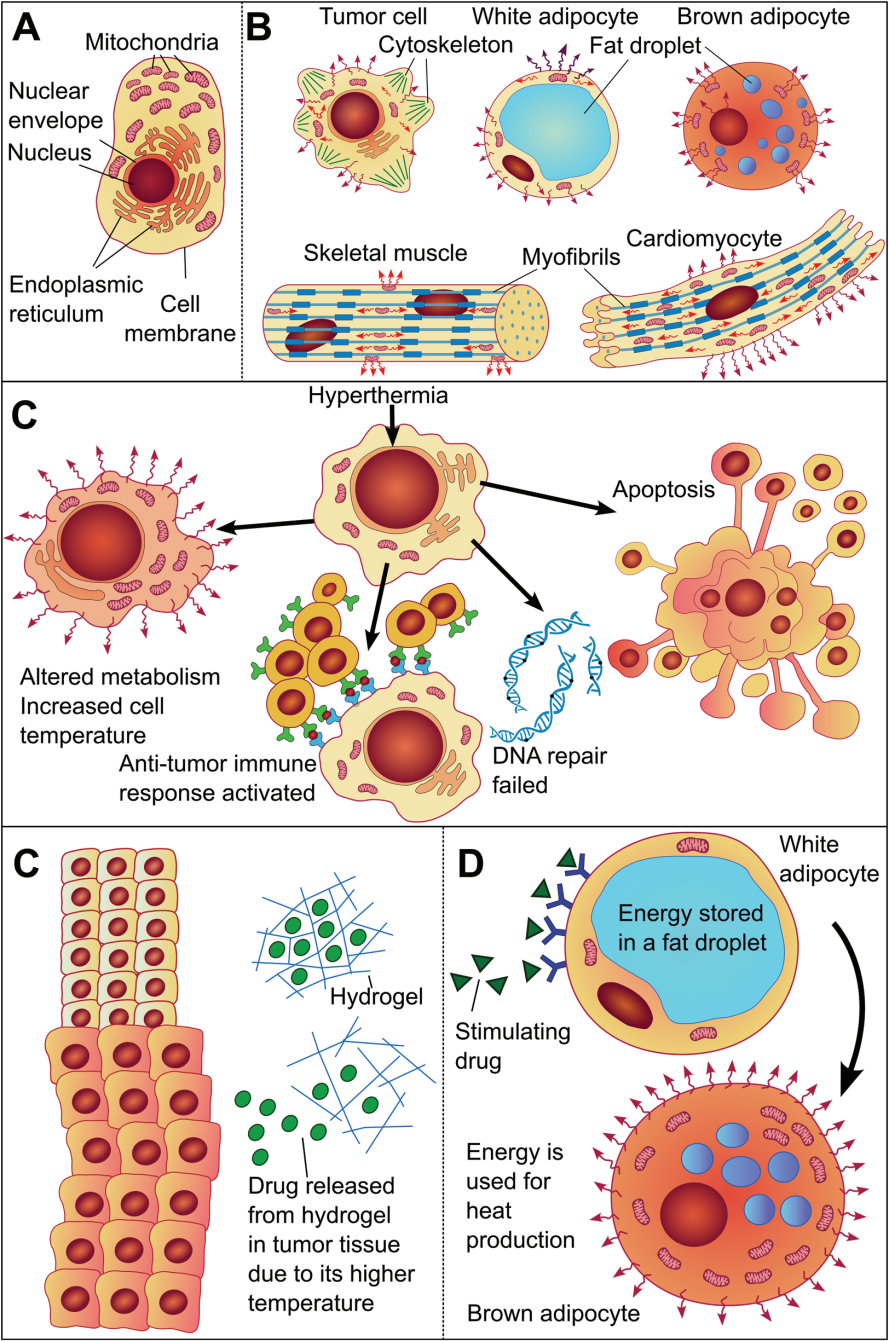
Heat production and dissipation in cells, the effect of temperature on cells, and selected therapeutic approaches. A) Cellular compartments most productive in heat generation–a scheme of a eukaryotic cell as a heat map. B) Cellular structures like cytoskeleton components in a tumor cell, fat droplets in adipocytes, or myofibrils in a cardiomyocyte or skeletal muscle significantly influence heat dissipation; arrows indicate the heat dissipations around these structures.^[^ ^14^ ^]^ C) Effects of hyperthermia on cancer cells: cancer cells treated with hyperthermia will change their‐ metabolism, and as a result, they will produce more heat, the recognition of cancer cells by cells of the immune system (cytotoxic T‐lymphocytes) will be strengthened, the DNA repair processes will be disturbed, apoptosis of tumor cells will be induced.^[^ ^30^ ^]^ D) The concept of selective tumor therapy: Antitumor drugs may be selectively delivered to tumors in hydrogels, which are released only in the tumor due to their higher temperature. E) The concept of potential treatment of obesity: Pharmacological stimulation of white adipocytes via β3‐adrenergic receptors could initiate their change to brown adipocytes, which will use the fat droplet for heat production.

Mitochondria,^[^ ^22^ ^]^ cell membrane,^[^ ^16^ ^]^ endoplasmic/sarcoplasmic reticulum,^[^ ^23^ ^]^ cell nucleus and its envelope connected to the reticulum^[^ ^24^ ^]^ represent the organelles with the most intensive thermogenesis (**Figure** **2**A).

Heat production inside the cell nucleus is associated with deoxyribonucleic acid (DNA) replication and transcription.^[^ ^25^ ^]^ The temperature changes inside the cells can influence the processes of cellular differentiation. For example, the application of intracellular thermometry led to the discovery that neuronal differentiation depends on intracellular thermogenesis associated with transcription and translation.^[^ ^26^ ^]^

Heat dissipation depends on the microanatomy of the cells. An undifferentiated cancer cell differs from that of an adipocyte from fat tissue or a muscle cell – cardiomyocyte.^[^ ^14^ ^]^ Cytosol facilitates heat dissipation, but even an undifferentiated cell with a high content of cytosol is 6× more resistant to heat transfer than water.^[^ ^27^ ^]^ Huge organelles like lipid droplets or myofibrils (fibrillar structures containing proteins actin and myosin ensuring muscle contractions) function as intracellular thermal insulators and reduce cellular heat conductivity^[^ ^14^ ^]^ (Figure 2B).

The progress in intracellular thermometry led to the formation of a new research field – thermal biology. High‐resolution intracellular temperature mapping detected thermogenic organelles and the thermogenic physiological functions. Thermal *signaling* was discovered, and temperature variations within biological cells serve as regulatory signals in biological pathways.^[^ ^28^ ^]^ Conversely, knowledge of cellular mechanisms stimulating thermogenesis in cells is essential for effectively testing newly developed polymeric nano thermometers and their transport into cells, for example, using endocytosis.^[^ ^29^ ^]^

## 3 Potential Therapeutic Regulations

The study of heat production, the formation of temperature gradients, and thermal dissipation at the level of cells and tissues is of considerable importance for a better understanding of cellular processes, but it also finds practical therapeutic use in oncology, pharmacology, and internal medicine (Figure 2C,D,E).

Therapies based on generating and managing localized heat are constantly evolving in oncology. Radiofrequency waves, ultrasound, and microwaves were explored in pre‐clinical and clinical settings, while nanotechnology‐based hyperthermia is not yet clinically established. The positive effects of hyperthermia, usually by heating tumor tissue to a temperature from ≈42 °C to ≈45 °C for ≈30 min to ≈60 min were repeatedly proven^[^ ^30^ ^]^ (Figure 2C).

The tumor vasculature is due to its rapid non‐physiological development immature^[^ ^31^ ^]^ and more sensitive to heat, which can cause endothelial damage, tumor tissue malnutrition, or blood stasis, leading to necrosis^[^ ^32^ ^]^ but sublethal heat treatment may also induce angiogenesis – new vessel formation. As a result, it causes a better supply of nutrients for the tumor.^[^ ^33^ ^]^ Contrarily, only a milder increase in temperature to ≈40 °C in the tumor can promote blood flow and better access to components of the immune system and drugs in tumor tissue.^[^ ^34^ ^]^ Cytotoxic lymphocytes play a crucial role in the anti‐tumor immune response. If the tumor cells are treated with hyperthermia, their expression of danger‐associated molecular patterns (DAMP) is elevated, and the cytotoxic lymphocytes can better target them and induce their programmed cell death – apoptosis.^[^ ^35^ ^]^

Therapeutic hyperthermia of tumors can overcome the natural thermo‐tolerance of cells ensured by specialized proteins called heat shock proteins (HSP), which are responsible for maintaining the folded state of proteins and repairing these folding malfunctions in the cell.^[^ ^36^ ^]^ Hyperthermia can affect cellular functions in multiple ways because the protein denaturation leads to the disruption of biochemical pathways, including transcription, replication, and DNA repair.^[^ ^37^ ^]^

The tumors' heat‐generating capacity and actual temperature are the functions of their vascularization, supplying the oxygen and nutrients for tumor metabolism and proliferation. These parameters can change dramatically during tumor development. The heat‐generating characteristics of tumors allowed the development of thermal imaging for their detection and diagnostics. It was documented that the average temperature of breast tumors is between ≈0.88  and ≈1.79 °C higher than that of the surrounding tissue.^[^ ^41^ ^]^ Elevated tumor temperatures were reported in numerous tumor types, including breast, bladder, lung, skin, and brain cancers.^[^ ^42^ ^]^ The functioning of hydrogels as potential carriers of therapeutic drugs is based on the increased temperature of tumors. The hydrogel carriers should release the drug only in the tumor microenvironment where the temperature is higher, thus enabling precise targeting of antitumor therapy. Proteins, peptides, or oligonucleotides can effectively be loaded into hydrogels without denaturation^[^ ^43^ ^]^ (Figure 2D).

Cells and tissues can adapt to changing environmental temperatures. White adipose tissue (WAT) serves as a storehouse of energy in the human body, while brown adipose tissue (BAT) can consume this energy in response to cold.^[^ ^44^ ^]^ WAT is located predominantly in subcutaneous and visceral regions and forms as much as 20 %–35 % of the body weight of a healthy person, and the amount of WAT increases in obesity.^[^ ^45^ ^]^ Initially, it was thought that BAT is present only in newborns, but it has also been discovered in adults in the cervical, supra‐clavicular, supra‐adrenal, and para‐spinal regions.^[^ ^46^ ^]^ The exposure of the organism to cold can activate either shivering thermogenesis involving ATP hydrolysis and heat production during striated muscle activity or non‐shivering thermogenesis in BAT, in which the production of ATP and heat in the mitochondria are disconnected (Figure 1A,B). Following the cold exposure, norepinephrine is released from the sympathetic nervous system, innervating BAT, and bound to β3‐adrenergic receptors of brown adipocytes. This signaling pathway results in uncoupling oxidative phosphorylation and production of ATP from heat production generated by transmembrane proton flow in the inner mitochondria membrane^[^ ^47^ ^]^ (Figure 1B). Specific fluorescent probes were designed to allow simultaneous detection of norepinephrine and intracellular temperature alterations.^[^ ^48^ ^]^

In contrast to studies performed on rodents, current literature rebates the relevance of β3‐adrenergic receptors in human BAT. β2‐adrenergic receptors were reported to play a crucial role in activating human BAT^[^ ^49^ ^]^ together with β1‐adrenergic receptors, which were found to be more abundant and active in human BAT.^[^ ^50^ ^]^

These findings have important implications for the development of pharmacological induction of these signaling pathways in WAT and BAT using agonists of adrenergic receptors to provide a potential treatment of obesity and comorbidities associated with it, such as hypertension or diabetes^[^ ^51^ ^]^ (Figure 2E).

Non‐shivering thermogenesis may serve as an example of the so‐called futile cycles. Generally, futile cycles are metabolic pathways that involve the simultaneous activity of opposing enzymes, leading to increased energy expenditure without synthesizing valuable products. Such a state was presumed to be a biological aberration and thus deemed an energy‐wasting “futile” cycle. However, evidence suggests that biological utilities emerge from futile cycles. In thermogenesis, these cycles play a crucial role in maintaining body temperature through adaptive thermogenesis and homeostasis. By promoting the cycling of substrates between anabolic and catabolic pathways, futile cycles can generate heat as a byproduct of metabolic activity.^[^ ^52^ ^]^

The best‐known futile cycle is the creatine cycle. Creatine acts as a rapid source of ATP through the phosphocreatine system, especially during short bursts of high‐intensity exercise. During periods of creatine loading, the enhanced availability of phosphocreatine allows for more significant ATP regeneration, which can lead to an increased caloric burn during physical activity. This heightened metabolic demand can stimulate thermogenesis as the body works to meet energy needs. When cycling off creatine, the body shifts back to its natural creatine production and storage levels, potentially adjusting metabolic efficiency and energy utilization. Numerous futile cycles function in the inter‐organ mode or different tissues. Pharmacological control of these cycles is a promising goal in treating metabolic disorders, with attention paid to body weight homeostasis.^[^ ^52^, ^53^ ^]^

The above‐given examples demonstrate the range of issues associated with studying cell temperature and its experimental and therapeutic regulation. They underline the importance of methodological approaches used for accurate temperature measurement at the level of individual cells and their organelles and the future development of new techniques.

## 4 Methods of Cell Temperature Measurement

Cell temperature measurement is essential for understanding cellular processes and their physiological and pathological stimuli responses. Over the years, several methods have been developed, each with its advantages and limitations. Broad categories include contact probes, offering direct but potentially invasive measurements; infrared thermography, which is non‐contact and useful for surface temperature mapping; and fluorescence‐based thermometry, providing high sensitivity and specificity, listed in **Table** **1**. Each method plays a critical role in studying cellular dynamics, contributing significantly to advancements in cellular biology, diagnostics, and therapeutic research. These evolving technologies continue to enhance our understanding of complex biological processes at the cellular level.

**Table 1:** Comparison of various methods for cellular temperature measurement.

Type	Method	Principle	Thermal resolution	Temporal resolution	Spatial resolution	Target	Ref.
Contact methods	Scanning Thermal Microscopy (SThM)	Thermal probe	–	–	–	Bamboo cells	[58]
	Thermocouple	Seebeck effect	100 mK	400 ns	–	U251 cell	[60, 61]
	Nanothermocouple probe	Seebeck effect	160 mK	0.2 s	–	Human liver cells (LO2) breast cancer cell (MCF7)	[55b]
	Thermoelectric Nanofluidic Probing	Temperature‐controlled ion migration	25 mK	0.9 ms	100 nm	SKOV3 cell	[55a]
Infrared (IR) Thermography	MIP microscope	Temperature‐dependent IR radiation	–	5 ms	500 nm	C. elegans	[66]
	Widefield photothermal sensing (WPS) microscope	Widefield photothermal sensing	–	1.1 µs	510 nm	SKOV3 Human ovarian cancer cell	[70]
	IR photothermal microscopy and Raman spectroscopy	IR photothermal microscopy and Raman spectroscopy	–	3 ms	290 nm	3T3‐L1 cell	[71]
Fluorescence Thermometry	Fluorescent proteins	Fluorescence polarization anisotropy of GFP	400 mK	20 ms	300 nm	HeLa and U‐87 MG cancer cell lines	[72]
	Fluorescent polymeric thermometer	Fluorescence lifetime imaging	180 mK	–	200 nm	COS 7 cell	[24]
	Fluorescent nanodiamonds	Shift of the zero‐field splitting parameter *D*_0_ using ODMR spectroscopy	600 mK	–	–	HeLa cells	[73]
	Organic dyes	Twisted intramolecular charge transfer	2.37%·°C^−1^	–	–	Hep‐G2 cells	[74]
	Quantum dots	Photoluminescent properties	1.79%·°C^−1^	–	–	NIH/3T3, HeLa cells	[75]
	Silver Nanowire	Adjusting the energy transfer between TR and the AgNWs	500 mK	–	–	HeLa cells	[76]

An important work was published ten years ago^[^ ^8^ ^]^ with a critical examination of temperature imaging techniques in single cells. Based on standard thermodynamic principles and scaling laws, the authors argued that thermogenesis in a single cell while being cultured in a water environment in a cell culturing dish can only locally increase temperature by up to ≈10 µK, which is not significant to be determined. This conclusion challenges the interpretations of recent studies that report temperature heterogeneities in single living cells, suggesting that the observed temperature variations reported in some studies are not physically feasible due to the limitations imposed by cells' natural heat dissipation processes. However, the employment of microcalorimetry can overcome this problem as the same amount of released energy not making any significant temperature change in large cell culturing dish volume can cause a considerable temperature increase in the microcalorimeter due to the limited volume of thermally isolated buffer volume.^[^ ^54^ ^]^

### 4.1 Contact Localized Temperature Measurement Techniques

Recent developments in localized temperature measurement within cellular studies have significantly advanced, integrating diverse principles of thermometry with sophisticated techniques.^[^ ^55^ ^]^ Key among these are thermocouples, PN and Schottky diode, and resistive temperature detectors (RTDs). These methods have been innovatively coupled with atomic force microscopy (AFM) tips, facilitating advancements in scanning thermal microscopy (SThM)^[^ ^56^ ^]^ for detailed applications.^[^ ^57^ ^]^

**Figure 3:**
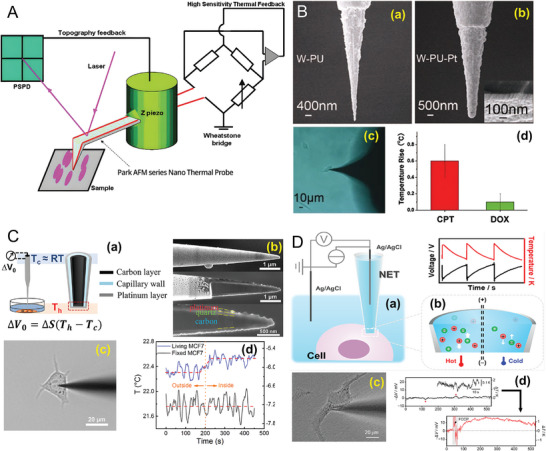
Contact localized temperature measurement techniques. A) Schematic illustration of an atomic force microscope (AFM) nano thermal probe system. This system utilizes a combination of topography and high‐sensitivity thermal feedback mechanisms. Topography is monitored through a position‐sensitive photodiode (PSPD) that detects laser deflections caused by surface irregularities as the probe scans the sample. The thermal feedback is achieved via a Wheatstone bridge circuit that measures the resistance changes in the probe, indicative of temperature changes due to thermal interactions with the sample. Reproduced with permission.^[^ ^58^ ^]^ Copyright 2019, Springer Nature. B) An SEM image of a tungsten‐polyurethane (W‐PU) nano thermal probe and its modification with a platinum layer (W‐PU‐Pt). The left image shows the W‐PU probe before platinum coating, with a scale bar representing 400 nm. The middle image shows the same probe after Pt coating, with a scale bar of 500 nm. The right image is a close‐up of the probe tip, displaying the platinum layer with a scale bar of 100 nm. Below these images is an image from an optical microscope showing the 2 probes during measurement and a graph (c) displaying the corresponding thermal feedback signal of 2 drug treatments. Reproduced with permission.^[^ ^60^ ^]^ Copyright 2011, Springer Nature. C) Panel C_a_: A schematic diagram of a micro‐thermal sensor based on a thermocouple principle where a voltage difference (ΔV_0_) is generated between 2 junctions at different temperatures (T_h_ and T_c_) due to the Seebeck effect. Panel C_b_: Cross‐sectional SEM image of a microcapillary thermal sensor with a carbon layer, capillary wall, and platinum layer. Panel C_c_: Brightfield microscope image of a living MCF7 cell being probed by the thermal sensor. Panel Cd: Graph depicting the temperature changes inside and outside of a living versus fixed MCF7 cell over time, accompanied by the respective voltage changes measured by the sensor. Reproduced with permission.^[^ ^55b^ ^]^ Copyright 2023, Royal Society of Chemistry. D) Schematic representation of a nano‐thermal electrochemical technique (NET) for measuring temperature changes at the cellular level. A cell is placed between 2 Ag/AgCl electrodes, and the temperature‐induced voltage changes (due to the Seebeck effect) are monitored over time, as shown in the graph above. The diagram illustrates cellular metabolic activity, highlighting heat (red) and cold (blue) areas. Below the schematic, a brightfield microscope image shows a cell positioned on the sensor. The lower graph displays the experimental data where the mitochondrial uncoupling agent FCCP is added to the system, shown by the arrow, leading to a change in temperature and corresponding voltage signal, indicating a change in metabolic activity. Reproduced with permission.^[^ ^55a^ ^]^ Copyright 2023, American Chemical Society.

The SThM, when integrated with atomic force microscopy (AFM) tips, achieves high‐resolution thermal imaging at the nanoscale.^[^ ^56a^ ^]^ This technique employs a nanoscale thermal probe, typically an AFM tip outfitted with thermocouples, diodes, or RTDs, to scan the cell surface (**Figure** **3**A) methodically. The unique feature of SThM lies in its capacity to precisely map thermal properties and heat flow within cells, a capability directly influenced by the curvature of the probe tip. SThM has become an invaluable tool in cellular research, distinguished by its precision in measuring cell temperatures. It operates through a nanoscale thermal probe that interacts with the cell surface, facilitating the detection of subtle temperature variations.^[^ ^58^ ^]^ This method enables researchers to define thermal characteristics at the cellular level, shedding light on the heat produced during various metabolic processes. The SThM technique enhances our understanding of cellular functions and metabolic activities by providing a means to visualize and quantify these thermal variations. It also aids in identifying irregularities in heat distribution within living systems. This detailed understanding is crucial for advancing research in cell biology, bioengineering, and medical science, as it opens new avenues for exploring the complex thermal dynamics that underlie the functioning of living organisms at a microscopic level.^[^ ^59^ ^]^

In addition to SThM, microscale thermocouples offer an alternative for temperature measurement in cellular studies. These thermocouples, capable of being directly inserted into tissues or cell cultures, provide localized temperature readings. Although highly precise, their invasiveness could potentially influence cellular behavior. Notably, a study developed a sensitive nanoprobe with a thermocouple metal junction (Figure 3B). This probe, consisting of W, PU, and Pt, features a tip with a sub‐500 nm radius, an angle from ≈10° to ≈20°, and a resistance from ≈500 to ≈1,500 Ω. Calibration showed a Seebeck coefficient from ≈6 to ≈8 µV°C^−1^, achieving ≈0.1 °C measurement precision.^[^ ^60^ ^]^ This setup was used to observe temperature fluctuations in lung epithelial cells, yielding insights into cellular responses.^[^ ^61^ ^]^ However, the complexity of such microfabrication techniques restricts the broader application of this method in single‐cell temperature sensing. The fundamental SThM technique was expanded using a 2ω method, improving its resolution.^[^ ^62^ ^]^

Fused silica or glass nanopipettes were employed to simplify the microfabrication as ideal for constructing nano thermocouple probes for single‐cell temperature sensing. The nanoscale heterojunction was created by coating different electrical materials on their surfaces.^[^ ^63^ ^]^ The nanopipettes made by pulling capillaries using laser irradiation are highly promising for intracellular analysis due to their cost‐effectiveness, adjustable nanoscale tips, and customizable surfaces.^[^ ^64^ ^]^ These nanopipettes can tune their tip's diameter to as small as ≈6 nm, minimizing cell disturbance. Their larger end can connect with a 3D microcontroller for versatile functions like cell insertion and scanning. A nano thermocouple probe is formed by depositing carbon and platinum separately on a fused silica nanopipette, showcasing excellent temperature resolution of ≈160 mK, rapid response of ≈0.2 s, and good stability (Figure 3C).^[^ ^55b^ ^]^ This advanced probe has been utilized to study intracellular temperature changes under drug effects and temperature gradients within 3D cell culture models, offering precise single‐cell temperature data with potential applications in drug screening, disease diagnosis, and treatment.

The nanopipette‐based electric thermometer (NET) was proposed as a superior alternative to traditional thermocouple probes for measuring intracellular temperatures in real‐time.^[^ ^55a^ ^]^ This innovative method utilizes temperature‐driven changes in ion movement within a solution, which result in altered ion behaviors and distributions. Researchers translate these changes into thermoelectric responses using a galvanostatic setup (Figure 3D). The NET's voltage‐temperature relationship demonstrates remarkable sensitivity, achieving a level of ≈11.1 mV K^−1^, significantly surpassing previous thermometric methods. Moreover, NET offers exceptional thermal precision at ≈25 mK, a high spatial resolution of ≈100 nm, a temporal resolution of ≈0.9 ms, and outstanding stability and reproducibility. These capabilities facilitate monitoring thermal fluctuations in stable cells and tracking heat dynamics during drug administration, overcoming challenges associated with earlier methods. The study using NET revealed thermal variations within individual cancer cells during immunotherapy, thus establishing a correlation between increased intracellular temperature and the enhanced survival and resistance of cancer cells in immunotherapeutic contexts.^[^ ^55a^ ^]^ This discovery provides a reliable tool for microscopic temperature monitoring and sheds light on immune evasion and therapeutic resistance mechanisms.

There are 2 fundamental problems with the probes regarding contact temperature measurement. First, the probes have to penetrate the cells physically, thus damaging them or at least touching the surface, causing potential cell damage. The second problem is the probe's thermal conductivity, introducing an error in the measurement.

### 4.2 Infrared (IR) Intracellular Thermography

Noncontact temperature measurement eliminates the problems related to contact measurement mentioned in the previous paragraph. However, it causes others with details described below.

Infrared thermography leverages the wavelength range of ≈8 µm to ≈14 µm to detect and interpret emitted infrared radiation from objects, converting this data into temperature readings. This method relies on the Stefan‐Boltzmann law,^[^ ^65^ ^]^ interpreting emitted infrared radiation from objects and converting this emission into temperature readings. Applied to intracellular temperature measurements, the technique deduces cell temperature by analyzing emitted infrared radiation, providing non‐invasive insight into the cell's thermal condition.

IR photothermal imaging is a technique used to detect IR absorption by optically probing thermal effects. Target molecules absorb mid‐IR wavelength and convert them into thermal energy, which causes localized changes in the refractive index. These changes affect the propagation of a visible probe beam, enabling detection. The resolution of this technique aligns with the diffraction limit of the visible probe's wavelength. A microscope using mid‐infrared photothermal (MIP) imaging was established, allowing the imaging of living cells and organisms for the first time. The achieved spatial resolution was ≈0.6 µm.^[^ ^66^ ^]^ The MIP imaging using a wavenumber of ≈2,100 cm^−1^ corresponding to the wavelength of ≈4.76 µm was demonstrated using difference frequency generation from femtosecond lasers.^[^ ^67^ ^]^ The spatial resolution was enhanced to ≈0.3 µm using a counter‐propagation system with a 532 nm laser and a water immersion objective. This enables the capture of a photothermal image of a single *E. coli* cell in the high‐wavenumber C‐H region.^[^ ^68^ ^]^ Photothermal phase‐sensitive lock‐in detection was combined with mid‐IR vibrational signatures to improve the contrast and spatial resolution for imaging single weakly absorbing features.^[^ ^69^ ^]^

**Figure 4:**
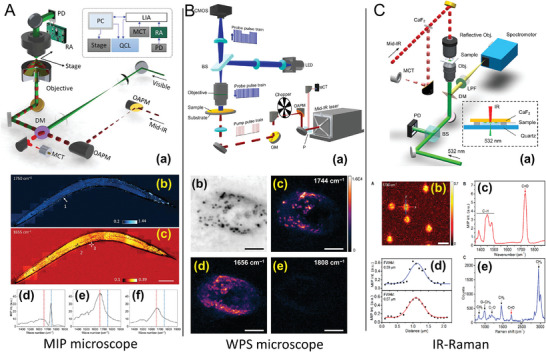
Three advanced microscopic techniques are used for temperature and bio‐imaging: mid‐infrared microscope (MIP), wide field photothermal sensing (WPS), and IR‐Raman. A) The MIP microscope setup shows a mid‐infrared quantum cascade laser (QCL) directed through an optical system to the sample, with a visible probe detecting photothermal effects, depicted in (a). Panels (b) and (c) display MIP images of a sample at different IR wavelengths, with corresponding intensity graphs below in (d), (e), and (f), showcasing the microscope's ability to discern chemical components within the sample. Reproduced with permission.^[^ ^66^ ^]^ Copyright 2016, AAAS. B) The WPS microscope is illustrated with its core components in (a), using blue and yellow beams for pulse train probing and thermal imaging. Panels (b), (d), and (e) present WPS images of different chemical components in a living SKOV3 human ovarian cancer cell cultured on a Si wafer highlighted at specific wavenumbers of 1,744·cm^−1^ (lipid), 1,656·cm^−1^ (protein), and 1,808 cm^−1^ (off‐resonance) demonstrating the detailed chemical mapping capabilities of the system. Reproduced with permission.^[^ ^70^ ^]^ Copyright 2019, AAAS. C) The IR‐Raman setup shown in (a) merges Raman spectroscopy and IR photothermal microscopy, using a 532 nm laser for dual functionality. Panel (b) features an IR‐Raman image with enhanced contrast, while (c) displays corresponding Raman spectra. The graph in (d) represents a photothermal profile across a sample, and (e) shows the detailed vibrational signature obtained through IRaman spectroscopy. Each panel demonstrates the respective technology's strength in providing high‐resolution, chemically specific imaging essential for understanding biological processes at the cellular and molecular levels. Reproduced with permission.^[^ ^71^ ^]^ Copyright 2012, American Chemical Society.Copyright 2019, American Chemical Society.

Label‐free visualization of biomolecules and materials within complex living systems has long been pursued through chemical contrast. IR spectroscopic imaging has made strides in this direction, but its application has been limited to dried tissues due to water's strong IR absorption. Furthermore, it suffers from low spatial resolution due to lengthy wavelengths and lacks optical sectioning capabilities. The authors addressed these constraints by utilizing a visible laser beam to sense the photothermal effect induced by vibrational absorption. They employed the MIP approach, which achieved a detection sensitivity of ≈10 mM with ≈500 nm lateral spatial resolution (**Figure** **4**A).^[^ ^66^ ^]^ This performance surpasses the diffraction limit of infrared microscopy, enabling label‐free 3D chemical imaging of live cells and organisms. The article uses MIP imaging to visualize endogenous lipid and exogenous drug distributions within single cells. Additionally, the authors successfully demonstrated in vivo MIP imaging of lipids and proteins in *Caenorhabditis elegans*. The technology has potential for various applications, including monitoring metabolic activities and high‐resolution mapping of drug molecules in living systems. These capabilities surpass the scope of current infrared microscopy.

In rapid IR absorption detection, traditional imaging modalities often struggle to track microsecond‐level thermal shifts, equivalent to roughly a million frames per second. The widefield photothermal sensing (WPS) microscope appears as an exciting innovation, boasting the ability to perform rapid chemical imaging at velocities reaching 1,250 frames s^−1^ (Figure 4B)^[^ ^70^ ^]^ to overcome this limitation. The WPS microscope utilizes a novel time‐gated detection technique with a pulsed illumination method initially devised for charting electronic currents in semiconductor circuits. Its groundbreaking feature is establishing a virtual lock‐in camera that coordinates exposure timing with the pulsation of the probe and infrared light at identical frequencies, thereby enabling precise timing control. This strategy facilitates the temporal mapping of fleeting thermal phenomena using conventional camera equipment, with the temporal resolution dependent on the pulse width of the probing beam. The applicability of the WPS microscope in biological exploration has been validated through its employment in capturing varied imaging of lipids, proteins, and off‐resonance frequencies in live SKOV3 ovarian cancer cells by adjusting the IR wavelength to 1,744 cm^−1^ for lipids, 1,656 cm^−1^ for proteins, and 1,808 cm^−1^ for off‐resonance imaging. This innovative technique marks a substantial leap forward in high‐speed thermal imaging.

The integration of IR photothermal microscopy and Raman spectroscopy, referred to as IRaman, is presented, utilizing a visible beam for both probing the photothermal effect and generating a Raman spectrum at the exact spatial resolution (Figure 4C).^[^ ^71^ ^]^ The system showcased the capability to produce IR photothermal images at specific IR wavelengths while conducting IR photothermal and Raman scattering spectroscopy at the point of interest within a sample, notably in the fingerprint region. This integrated setup offers complementary chemical information for complex biological specimens by capitalizing on the distinct sensitivities of Raman and IR to different vibrational modes. The IRaman microscope was employed to visualize and analyze individual lipid droplets in a white adipocyte differentiated from mouse embryonal cell line 3T3‐L1. The MIP image, as opposed to the reflection image of the 3T3‐L1 cell, demonstrated the capacity to distinguish LDs from other subcellular structures like the nucleus within the 3T3‐L1 cell.

The primary advantage of IR thermography is its non‐contact nature, which allows for temperature measurement without any physical interference with the cell, which is crucial in preserving the natural state of the biological specimen. Additionally, it will enable the ability to visualize and measure temperature distribution over the entire cell or tissue surface, allowing the cell surface thermal mapping. One significant limitation is its spatial resolution, which may not be sufficient for detecting minimal temperature changes at the cellular level. The accuracy of temperature readings can also be affected by various environmental factors such as humidity, air currents, and other IR sources. Moreover, this method measures surface temperature, which may not accurately represent the internal temperature of the cell, especially if the cell has varying internal structures or is undergoing dynamic metabolic processes. IR thermography offers a unique approach to measuring cell temperature by detecting IR radiation and avoiding direct contact with the cell. While it provides valuable insights into thermal patterns and can measure temperature changes over a large area, its resolution and environmental sensitivity must be considered when interpreting results. The method is beneficial in studies where non‐invasive temperature measurement is crucial, and knowledge of surface temperature distributions is of interest.

### 4.3 Fluorescence Thermometry

Fluorescence thermometry for intracellular temperature measurement utilizes temperature‐sensitive fluorescent materials that change their emission properties in response to temperature variations.^[^ ^29^ ^]^ This technique enables precise monitoring of cellular or even subcellular temperature changes. It is particularly effective in studying temperature‐dependent biological processes and cellular responses to environmental changes. Unfortunately, a fundamental problem exists, as introducing a foreign substance inside the cells alters its properties. Nevertheless, recent advances in this field include the development of various fluorescent materials, such as green fluorescent proteins (GFP),^[^ ^72^ ^]^ fluorescent polymeric thermometers (FPTs),^[^ ^24^ ^]^ fluorescent nanodiamonds (FND),^[^ ^73^ ^]^ fluorescent organic dyes (FOD),^[^ ^74^ ^]^ quantum dots (QD),^[^ ^75^ ^]^ and silver nanowires (AgNWs),^[^ ^76^ ^]^ enhancing the sensitivity and spatial resolution of temperature measurements within cells. This method's ability to provide real‐time, non‐invasive temperature data makes it invaluable in cellular biology and medical research.

**Figure 5:**
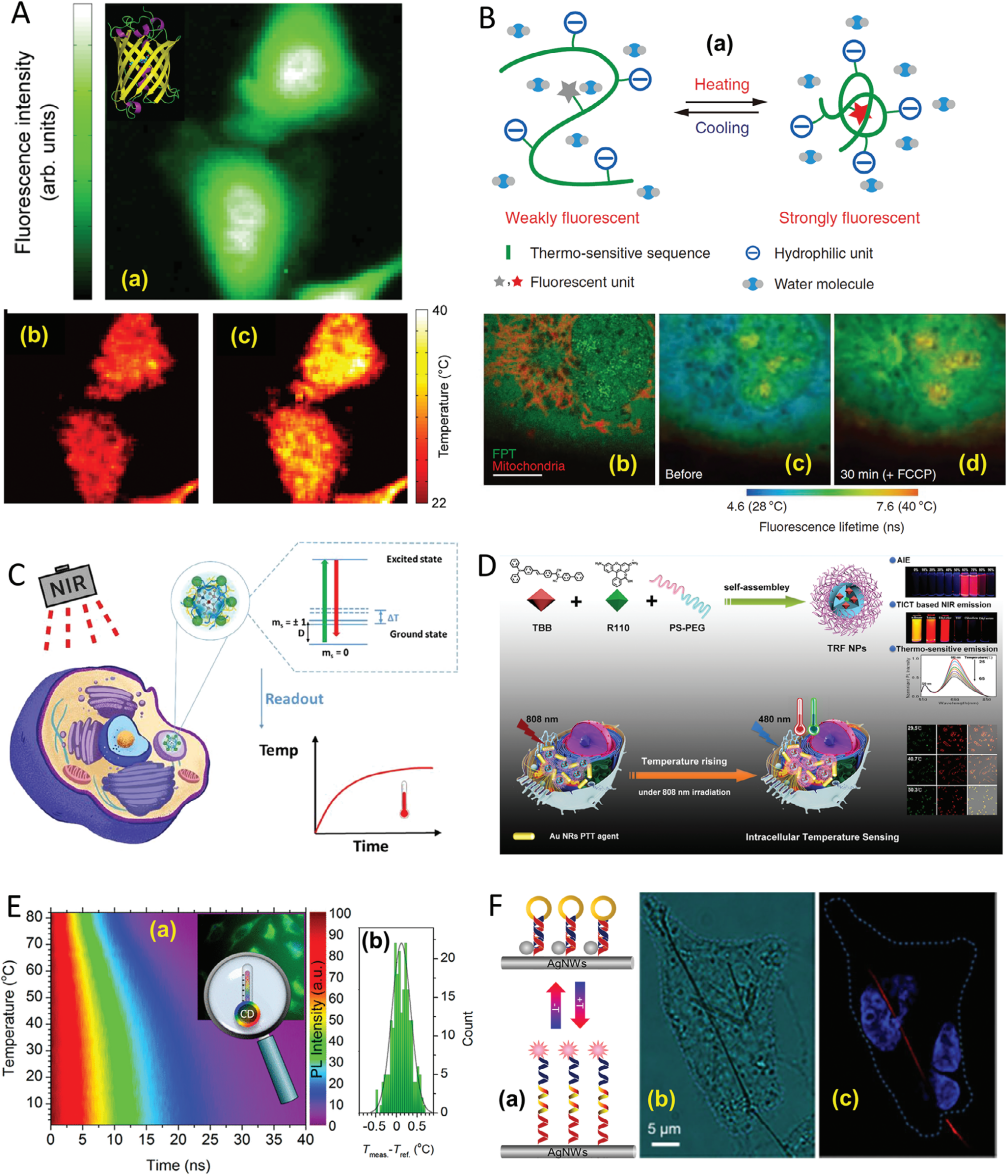
Fluorescence‐based cell thermometry. A) Fluorescence intensity of GFP transfected HeLa cells and a 3D representation of the structure of GFP, and the temperature map of no heating and heating the NR with a focused infrared laser with a power of ≈50 mW, located ≈50 µm to the right of the center of the image. Reproduced with permission^[^ ^72^ ^]^ Copyright 2012, American Chemical Society. B) Functional diagram of FPT in an aqueous medium for intracellular temperature mapping. Temperatures increase near the mitochondria after the inhibition of ATP synthesis by the uncoupler FCCP. Confocal fluorescence images of FPT (green) and MitoTracker deep red FM (red; left) and fluorescence lifetime images of FPT (middle and right). Reproduced with permission.^[^ ^24^ ^]^ Copyright 2012, Springer Nature. C) Schematic presentation of the temperature measurements in living cells. A simplified energy level diagram of the NV−center in ND displays the ground state of the spin‐triplet and the excited state. The shift of the zero‐field splitting parameter D0 can monitor the intracellular change in temperature by optically detected magnetic resonance spectroscopy. Reproduced with permission.^[^ ^73^ ^]^ Copyright 2021, American Chemical Society. D) The principle of the fluorescent organic dyes, TRF NPs, is used for intracellular temperature measurement. The TRF NPs showed a temperature sensitivity of ≈2.37 % °C^−1^, comprehensive temperature response ranges from 25 to 65 °C, and excellent temperature‐sensitive emission reversibility. Reproduced with permission.^[^ ^74^ ^]^ Copyright 2020, American Chemical Society. E) The intracellular nanothermometers using water‐soluble N, S‐co‐doped carbon dots (CDs) based on temperature‐dependent photoluminescence lifetime. The CDs exhibit excellent water dispersibility, superior photo‐ and thermostability, extraordinary environment, concentration independence, high storage stability, and reusability. Reproduced with permission.^[^ ^75^ ^]^ Copyright 2017, American Chemical Society. F) Rationale of TR‐DNA−AgNW response to temperature as well as bright field image of the monofilament TR‐DNA−AgNW in a HeLa cell at room temperature and its fluorescence images 26 °C with 405 and 561 nm laser as excitation. Reproduced with permission.^[^ ^76^ ^]^ Copyright 2018, American Chemical Society.

Due to their temperature‐sensitive fluorescence properties, GFPs are employed as thermal nanoprobes for intracellular temperature mapping. This method involves tracking changes in fluorescence polarization anisotropy of GFP in response to temperature variations, enabling high‐resolution temperature measurements within cells. The approach achieved a spatial resolution of ≈300 nm and temperature accuracy of ≈0.4 °C successfully applied to GFP‐transfected HeLa and U‐87 MG cancer cell lines (**Figure** **5**A).^[^ ^72^ ^]^ The heat delivery was monitored by photothermal heating of gold nanorods near the cells. The primary advantage of using GFP as a thermal nanoprobe is its non‐invasiveness and compatibility with live cell imaging, which holds significant promise for fundamental research and applied studies in fields ranging from molecular biology to therapeutic and diagnostic endeavors. However, it may have limitations in quantification accuracy and sensitivity to environmental factors like pH changes or photobleaching.

The FPTs have emerged as a novel method for intracellular temperature measurements, offering high sensitivity and spatial resolution. These thermometers utilize a thermo‐responsive polymer combined with an environment‐sensitive fluorophore, exhibiting a change in fluorescence lifetime in response to temperature variations within the cell, making it possible to map intracellular temperature distributions with high precision. One of the main advantages of FPTs is their ability to provide temperature measurements independent of probe concentration or environmental factors like surrounding proteins or viscosity. The intracellular temperature mapping using an FPT combined with fluorescence lifetime imaging microscopy has been demonstrated, achieving spatial and temperature resolutions at the diffraction‐limited level to ≈200 nm and within a range from ≈0.18 to ≈0.58 °C (Figure 5B).^[^ ^24^ ^]^ This innovative approach revealed that in a COS‐7 cell, the nucleus and centrosome exhibited temperatures significantly higher than the cytoplasm, with the temperature difference varying with the cell cycle. Additionally, heat production from mitochondria was observed, manifesting as localized temperature increases near these organelles. One such limitation is the potential for a more significant error in fluorescence lifetime determination at higher temperatures, which may reduce the accuracy of thermometry. A time‐correlated single photon counting system‐based fluorescence lifetime imaging microscopy (FLIM) further enhances this method,^[^ ^77^ ^]^ enabling precise temperature‐dependent fluorescence lifetime measurements of FPT. This advanced thermometric methodology provides high spatial and temperature resolution and has been instrumental in uncovering novel biological insights, such as intracellular temperature gradients and organelle‐specific thermogenesis.

Fluorescent nanodiamonds (FNDs) are emerging as effective thermal nanoprobes for intracellular temperature mapping using their nitrogen‐vacancy (NV) centers. Their unique ability to measure temperature with high spatial and temporal resolution benefits the intracellular environment. The FNDs method detected temperature variations of less than 1°C within cells, providing insights into localized thermogenesis near organelles like the nucleus and mitochondria.^[^ ^28^ ^]^ FNDs leverage their fluorescence properties, which change in response to temperature variations. The FND have been developed as nanoscale temperature sensors, independent of external conditions like pH or ions. These FND nanothermometers are coated with a nanogel shell and integrated with indocyanine green, serving as heat generators and sensors (Figure 5C). They have demonstrated the ability to precisely induce and simultaneously record apoptosis and temperature increases in cancer cells.^[^ ^73^ ^]^ The nanodiamond‐based approach provides insights into local temperature changes within cells, revealing that cells can tolerate significant temperature increases without impacting viability. This advancement is crucial for exploring photothermal effects in different cellular environments and their impact on cell viability. Moreover, it opens new avenues for studying temperature‐driven biological processes at the intracellular and subcellular levels, highlighting the potential of FND nanothermometers in biological and medical research.

FPT, combined with the FND method, was used to measure intracellular temperature^[^ ^26^ ^]^ to find that intracellular temperature variations influence neuronal differentiation by focusing on neurite outgrowth. FPTs monitored changes in fluorescence lifetime, while FNDs measured temperature shifts through optically detected magnetic resonance spectra. Additionally, localized intracellular heating was achieved using an IR laser. The study reveals that increased local temperature, especially in the nucleus, promotes neurite outgrowth, suggesting that intracellular thermogenesis is critical for neuronal differentiation. This research underscores the importance of precise temperature measurement in understanding cellular processes and could lead to new therapeutic strategies targeting temperature‐driven cellular behaviors.

Fluorescent organic dyes (FODs), such as Rhodamine B, Fluorescein, and BODIPY, are widely used for intracellular temperature mapping due to their distinctive fluorescence characteristics. These dyes change their emission properties in response to temperature variations within cells. The advantage of fluorescent organic dyes is their high sensitivity and ability to provide real‐time, dynamic temperature measurements at the cellular level. A radiometric fluorescent thermometer, encapsulating near‐infrared (NIR) fluorophore TBB and rhodamine 110 in TBB&R110@F127 nanoparticles (TRF NPs), exhibited a sensitivity of 2.37 %·°C^−1^ and a comprehensive response range (Figure 5D). The TRF NPs were applied to the human cell line Hep‐G2, derived from hepatocellular carcinoma Hep‐G2 cells, and effectively monitored temperature variations from 25 to 53 °C.^[^ ^74^ ^]^ These dyes offer sensitive and real‐time measurements, but their utilization is limited by photobleaching, phototoxicity, and the influence of environmental factors and dye concentration on accuracy.

The principle of using quantum dots (QDs) for intracellular temperature mapping hinges on their photoluminescent properties, which shift with temperature changes, allowing for cellular temperature mapping. A technique using water‐soluble S and N co‐doped carbon dots (N, S‐CDs) demonstrates temperature‐dependent photoluminescence lifetimes, with lifetimes consistent across pH values from ≈5 to ≈12, CDs concentrations ranging from ≈1.5 × 10^−5^ to ≈0.5 mg·mL^−1^, and in environments with up to ≈0.7 mol L^−1^ of NaCl (Figure 5E). These CDs are biocompatible, non‐toxic, and exhibit remarkable photostability and thermostability, with their performance validated in cell viability and flow cytometry analyses using NIH/3T3 and HeLa cell lines. Their consistent performance in temperature measurements between ≈15 and ≈45 °C was observed over multiple experiments, highlighting their potential in biomedical applications.^[^ ^75^ ^]^ However, using QDs for biological applications must consider potential cytotoxicity and the challenge of precisely correlating fluorescence changes with specific temperature measurements.

The use of silver nanoparticles (AgNWs) to create a nanosized fluorescence thermometer represents a novel method for accurately measuring intracellular temperatures with impressive spatial resolution.^[^ ^76^ ^]^ This thermometer is ingeniously crafted by affixing thermally sensitive DNA stem loops labeled with Texas red fluorophore onto AgNWs (Figure 5F). Temperature fluctuations alter the TR‐DNA stem‐loop structure, modulating the energy transfer to AgNWs and changing the fluorescence intensity. This method exhibits a sensitivity range from ≈30 to ≈40 °C, with a precision of ≈2.6 % °C^−1^, and has been effectively used to detect temperature changes in individual cells via laser confocal microscopy.

Recent research introduces fluorescent polymer probes capable of simultaneously monitoring mitochondrial temperature and ATP fluctuations in living cells.^[^ ^15^ ^]^ The probes, designed to target mitochondria, respond to temperature and ATP changes with distinct fluorescent signals, providing valuable insights into the relationship between heat and energy production in cells. This dual‐sensing approach enhances our understanding of cellular metabolism and energy dynamics, offering significant implications for studying mitochondrial function in health and disease. It can also simultaneously detect temperature as well as Ca^2+^ concentration during intracellular heat production.^[^ ^20^ ^]^

The detection mechanism of the dual‐function fluorescent probe involves a thermosensitive polymer, poly(N‐isopropyl acrylamide) (PNIPAm), combined with a polarity‐sensitive fluorescent dye, AANBD, and a norepinephrine (NE)‐selective sensor.^[^ ^48^ ^]^ PHE. The PNIPAm polymer responds to temperature changes by transitioning from a hydrophilic to a hydrophobic state, which alters the fluorescence intensity of the AANBD dye, enabling precise temperature detection. Simultaneously, the PHE moiety binds explicitly to NE through interactions with the primary amino group and the phenolic hydroxyl group of NE, leading to a fluorescence change that allows for selective NE detection. This dual‐sensing capability enables real‐time monitoring of intracellular temperature and NE levels, making it a powerful tool for studying neurotransmitter dynamics, particularly in understanding neurotransmitter reuptake mechanisms and their implications in neuropsychiatric conditions such as depression.

In recent years, the method of fluorescence bleaching independent temperature measurements based on DNA melting temperature has been demonstrated as a precise method of temperature monitoring^[^ ^78^ ^]^ and determining temperature uniformity.^[^ ^79^ ^]^ Unfortunately, that technique is suitable for higher temperatures than the cell can tolerate; thus, it must be modified to measure the cell temperature.

### 4.4 Other Chemical Probe‐Based Techniques of Intracellular Temperature Measurement

Recently, the development and application of luminescent molecular thermometers for ratiometric sensing of intracellular temperature was discussed.^[^ ^80^ ^]^ These thermometers exhibit temperature‐dependent emission properties and are valuable tools for measuring the temperature of tiny spaces within living cells. The authors emphasize that intracellular temperature is closely linked to various cellular processes, including gene expression, enzyme activity, metabolism, and disease progression. Accurate intracellular thermometry can provide critical insights into these processes and potentially lead to novel diagnostic and therapeutic approaches. The work covers different types of luminescent molecular thermometers, focusing on those that measure the emission intensity ratio at 2 different wavelengths, which is particularly suitable for precise and accessible intracellular temperature measurements. These thermometers are small enough to enter cells without invasive procedures and can offer high temporal resolution. The review highlights several ratiometric luminescent molecular thermometers based on small organic molecules, polymeric materials, and fluorescent proteins, each offering varying sensitivity and temperature resolution levels.

In recent years, the chemiluminescence method has been developed using thermoresponsive polymer nanocomposite PFLNC@aptamer to target ATP and measure intracellular levels. It monitored the chemiluminescence at different temperatures, improving enzymolysis efficiency by 21% and achieving a detection limit of 3.3 nM. This nanocomposite effectively avoids interference during delivery, ensuring reliable ATP detection for practical applications.^[^ ^17^ ^]^

### 4.5 Other Techniques of Localized Temperature Measurement

**Figure 6:**
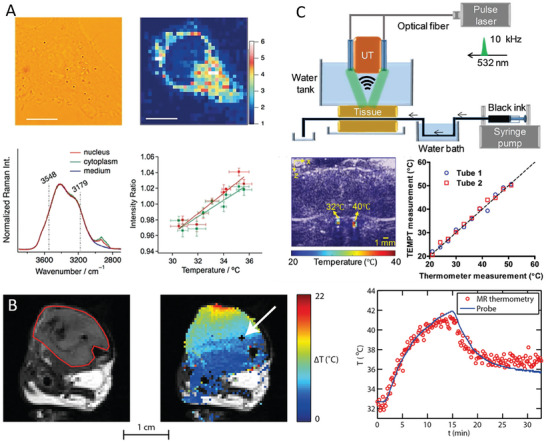
Other techniques of localized temperature measurement. A) The bright‐field image of a single HeLa cell and its Raman image at 750 cm^−1^ shows the distribution of cytochrome in a cell. Representative Raman spectra of the nucleus, cytoplasm, and Hanks balanced salt solution (HBSS) medium in the O‐H stretching and C‐H stretching bands region. Plots of the Raman intensity ratios (I_3548_/I_3179_) of the nucleus and cytoplasm at different HBSS temperatures show the temperature dependence of the O‐H stretching Raman band of HeLa cells. Reproduced with permission.^[^ ^81^ ^]^ Copyright 2020, Wiley‐VCH Verlag GmbH. B) An anatomical reference image of the axial cross‐section of a mouse hind limb with a red contour indicates the outline of the tumor. The temperature map obtained after ≈13 min of laser‐induced heating shows a substantial temperature increase at the top of the tumor, where the light entered the tissue. The MRT data in a pixel next to the probe followed the probe temperature with a root mean squared error of ≈0.74°C, calculated over the entire experiment duration. Reproduced with permission.^[^ ^83^ ^]^ Copyright 2017, SPIE. C) Schematic of the photoacoustic tomography system. Two ink‐filled tubes with different temperatures were sandwiched between 2 slices of chicken tissue (each ≈1.5 cm thick). Thermoacoustic Imaging (shown in color) overlaid onto the ultrasound image (gray) of the 2 tubes with measured temperatures of ≈32 and ≈40°C, respectively. The temperature of the 2 tubes was measured using the thermoacoustic method as a function of the reference temperatures measured by the needle thermometer. Reproduced with permission.^[^ ^85^ ^]^ Copyright 2019, Optical Society of America.

Several less traditional and innovative methods for localized temperature measurement inside living cells are being explored. These methods have been developed to overcome the limitations of conventional techniques like thermocouples, infrared thermography, and fluorescence thermometry. Here's a list of these alternative methods:

1. *Raman Spectroscopy*: This technique uses inelastic light scattering (Raman scattering) to measure a system's vibrational, rotational, and other low‐frequency modes. In cells, Raman spectroscopy can determine temperature changes based on shifts in vibrational energy levels of certain molecular bonds. Raman microscopy, mainly through the analysis of the O‐H stretching bond of water, offers a label‐free method for simultaneous intracellular and extracellular temperature measurement (**Figure** **6**A).^[^ ^81^ ^]^ This technique leverages the temperature‐dependent changes in the Raman bond of water to create a calibration curve. Analysis of Raman spectra at different temperatures found that the intensity ratio of specific regions of the O‐H stretching bond correlates linearly with temperature changes. This method is advantageous as it avoids photobleaching and phototoxicity, is applicable across all cell areas due to the ubiquity of water, and can measure extracellular temperatures concurrently. Furthermore, its insensitivity to other cellular conditions like pH and the ability to provide additional molecular information make it a versatile tool for biological analysis.
2. *Magnetic Resonance Thermometry (MRT)*: The MRT is an essential non‐invasive technique in medical diagnostics and research, harnessing the temperature‐dependent properties of water proton resonance frequency or diffusion coefficients.^[^ ^82^ ^]^ Particularly advantageous for deep tissue analysis, MRT visualizes 3D temperature distribution, which is crucial for understanding biophysical and physiological processes. Over the past 2 decades, its evolution has significantly enhanced its accuracy and robustness for in vivo applications, primarily focusing on relative temperature mapping. The ongoing efforts aim to achieve quantitative temperature readings, which are vital for evaluating thermal therapy efficacy and setting new benchmarks in diagnostic and therapeutic applications. MRT's primary application has been in tissue thermal imaging (Figure 6B)^[^ ^83^ ^]^ and intratumoral temperature measurements during thermal therapy.^[^ ^84^ ^]^
3. *Thermoacoustic Imaging*: This hybrid imaging technique combines ultrasound and electromagnetic energy.^[^ ^86^ ^]^ When tissues are exposed to a pulsed electromagnetic field, thermoacoustic waves are generated due to thermal expansion, which can be detected and used to infer temperature variations. Combines ultrasound and electromagnetic energy to detect thermoacoustic waves generated by tissue thermal expansion. This method allows for temperature mapping and is helpful in medical diagnostics and cellular studies (Figure 6C).^[^ ^85^ ^]^ However, its resolution and sensitivity may be limited compared to other imaging techniques.

Comparatively, Raman spectroscopy stands out for its non‐invasiveness and label‐free approach, while methods like MRT and thermoacoustic imaging excel in deep tissue analysis. Nanoparticle‐based sensors and microwave thermometry offer high precision but might face biocompatibility and cellular interference challenges, respectively. Microfluidic devices provide an excellent platform for controlled experiments but may be limited in spatial resolution. Each method's applicability depends on the specific requirements of the biological study. Each technique offers unique advantages and potential applications in cellular biology, medical diagnostics, and therapeutic monitoring. They represent the cutting‐edge research in cellular temperature measurement, evolving to provide more accurate, less invasive, and higher‐resolution temperature data within living cells.

### 4.6 A Technique for Global Cell Temperature Measurement

Extensive studys on the development of ultrasensitive microcalorimeters and their application in bioanalysis and energy balance monitoring have been recently published.^[^ ^54b^ ^]^ It elaborates on advanced microfabrication techniques that enable these devices to detect minute heat changes at the single‐cell or even subcellular level, with high sensitivity and resolution. The paper explores various designs of microcalorimeters, their fabrication, the challenges faced, and the potential for future innovations in the field. The applications discussed in the manuscript range from cell metabolism research to biomolecule interactions and calorimetric detections, highlighting the versatility and importance of microcalorimeters in contemporary bioresearch.

**Figure 7:**
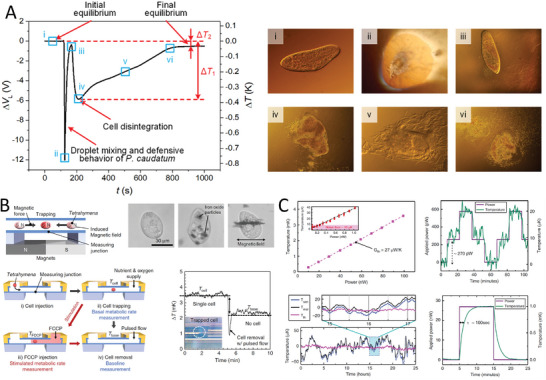
Experimental setups and results for studying microorganism responses to physical stimuli or changes: A) The thermodynamic response of *P. caudatum* to added surfactant showing ΔV_L_ as a function of t, where ΔV_L_ is proportional to ΔT of the cells. The initial equilibrium is disrupted, leading to cell disintegration, marked by a steep decline in ΔT. After a transition phase involving droplet mixing and defensive behavior, the *P. caudatum* reaches a final equilibrium by eliminating the metabolism of all *P. caudatum* by destroying them. ΔT_2_ value represents the metabolic activity of *P. caudatum*. Reproduced with permission.^[^ ^54a^ ^]^ Copyright 2021, Elsevier. B) Structure and operation of the microfluidic chip calorimeter using magnetic cell trapping for single‐cell measurement. The curve showed temperature change over time during these steps, with insets showing a single trapped cell and the effect of cell removal on temperature. Reproduced with permission.^[^ ^87^ ^]^ Copyright 2020, Springer Nature. C) The performance of the calorimeter monitors the metabolic heat output of individual *C. elegans*, including G value, power resolution, long‐time temperature stabilization, and time constant. Reproduced with permission.^[^ ^88^ ^]^ Copyright 2020, Springer Nature.

A droplet‐based differential microcalorimetric system (**Figure** **7**A) for real‐time energy balance monitoring of the protist, *Paramecium caudatum* (*P. caudatum*) was recently introduced.^[^ ^54a^ ^]^ This system demonstrated the microcalorimeter's capability to dynamically monitor temperature change during the organism's defensive behavior with temperature and power resolution of ≈49.7 µK and ≈14 nW, respectively. Then, using the heat balance equation,^[^ ^65^ ^]^ the authors calculated the released energy of a single cell exocytosis event of ≈0.75 mJ during the *P. caudatum* defense process. The findings shed light on the energy consumption during the defense mechanism, offering insights into the metabolic heat of protist metabolism and potentially other cellular processes. This platform provides a new perspective on studying cellular energy dynamics non‐invasively.

A microfluidic chip calorimeter, with a sensitivity of ≈200 pW, has shown significant improvement over previous technologies by measuring the metabolic rate of *Tetrahymena thermophila* as an example (Figure 7B).^[^ ^87^ ^]^ This device employs a suspended microfluidic channel in a vacuum for single‐cell metabolic heat measurement and real‐time monitoring of metabolic stimulation by mitochondrial uncoupling agents. The results demonstrate a correlation between metabolic rate and cell size, suggesting potential biological research and medical diagnostics applications.

A highly sensitive calorimetric platform (Figure 7C) has been designed to measure the metabolic heat output of individual *Caenorhabditis elegans* (*C. elegans*) worms with a resolution of ≈270 pW.^[^ ^88^ ^]^ This marks a significant advancement over previous technologies and allows for real‐time metabolic measurements across different life stages of the worms. The study shows that metabolic output is lower in long‐lived daf‐2 mutants of *C. elegans*, indicating the potential of this tool in studying metabolism‐related disease development and aging. The calorimeter integrates a fluidic environment and optical imaging, offering detailed insights into the thermodynamics of biological processes in model organisms and single cells.

Despite their high sensitivity, capable of determining temperature changes at 10 µK or below, calorimeters face several issues. They can only determine global temperature changes, missing local temperature variations and potential hot spots in cells. Furthermore, the ultimate power resolution of a microcalorimeter is a function of its thermal conductivity (*G*).^[^ ^89^ ^]^ A lower *G* value means better power resolution and a higher thermal time constant, slowing an ultrasensitive system. This issue can be mitigated by reducing the microcalorimeter's thermal capacitance. However, there is a limit to this approach since cells require a certain amount of buffer solution and tend to proliferate in colonies rather than as individual cells.

Different microcalorimeter systems have previously been used to enhance temperature response and monitor the energy balance of living organisms, such as the detection of exocytosis of trichocytes, i.e., the defense behavior of *P. caudatum*,^[^ ^54a^ ^]^ as well as the metabolic heat rates of *Tetrahymena thermophila* ^[^ ^87^ ^]^ and the metabolic activity of individual *C. elegans*.^[^ ^88^ ^]^ However, there is still ample room for improvement in this area.

## 5 Cellular Temperature Measurement: Advances, Challenges, and Future

### 5.1 Challenges and Limitations

Addressing the challenges and limitations in measuring and modulating cell temperature is essential for advancing our understanding of cellular processes and developing therapeutic applications. Achieving the necessary spatial and temporal resolution for accurate temperature measurement is a primary challenge in this field. Classic methods like thermocouples^[^ ^60^ ^]^ and infrared thermometry^[^ ^90^ ^]^ are reliable and well‐established but often fall short of providing the detailed resolution required at the cellular level. Furthermore, these methods might not capture rapid temperature fluctuations within cells, a crucial aspect of understanding dynamic cellular processes. Advanced techniques such as fluorescence thermometry^[^ ^91^ ^]^ and SThM bring improvements in the resolution.^[^ ^56a^ ^]^ However, these methods introduce challenges, including the need for sophisticated instrumentation^[^ ^92^ ^]^ with a temperature‐controlled fluid cell for cell seeding and culturing^[^ ^93^ ^]^ and complex procedures. Such requirements contribute to the technical complexity and escalate the costs associated with these methods, thereby limiting their widespread use in research settings.

Accuracy and sensitivity in temperature measurement are other significant issues. In non‐contact methods like IR thermography, external environmental factors such as surface emissivity^[^ ^65^ ^]^ and angle of IR incidence can significantly influence the readings, raising questions related to the accuracy of the obtained data. Advanced techniques like fluorescence thermometry, while providing more sensitive measurements, still face challenges in detecting subtle temperature changes within cells. The accuracy of these measurements is crucial for correctly interpreting cellular responses to various stimuli. Furthermore, the invasiveness of specific measurement techniques, particularly those that require direct contact with the cells, can be a concern. These methods can alter cell behavior or affect cell viability, which may skew the results or limit the technique's applicability. Non‐contact methods offer a less invasive alternative, but sometimes, at the cost of reduced precision, external environmental factors can still influence them.

The technical complexity and cost associated with advanced cell temperature measurement techniques are significant barriers to their adoption in broader research applications. Techniques such as quantum dot thermometry and SThM involve complex procedures and high expenses, which can be excessive for many research laboratories. These methods offer advanced capabilities, but their practicality and accessibility are limited.

In summary, while significant progress has been made in the methods for measuring and modulating cell temperature, substantial challenges still need to be addressed. These include improving resolution and sensitivity, reducing the invasiveness of measurement techniques, and overcoming technical and cost barriers. Continued innovation and research in this field are essential for developing more effective and accessible methods for studying cellular thermodynamics and translating these findings into practical therapeutic applications.

### 5.2 Technological Advances and Future Directions

Cell temperature measurement and modulation have seen remarkable technological advancements in recent years, signaling a bright future for research and potential applications. These advancements enhance our understanding of cellular processes and pave the way for novel therapeutic methods.

The critical problem of the cell temperature measurement is the biophysics of thermogenesis, as it was calculated that the local temperature increase should be only 10 µK or lower^[^ ^8^ ^]^ with an assumption that the cells are located on conventional cell culturing dishes. This temperature increase can be enhanced by employing a microcalorimeter, where the cells are cultured in a confining environment with a total volume of buffer solution in nL.

The domain of cell temperature regulation has made significant technological strides, auguring well for both scientific inquiry and practical applications. These advancements deepen our grasp of cellular mechanisms and herald innovative treatments. A substantial hurdle in cell temperature assessment relates to thermogenesis biophysics, predicting minimal temperature rise.^[^ ^8^ ^]^ Microcalorimeters in confined volumes show promise in enhancing this detection. High‐resolution techniques, such as fluorescence thermometry and SThM, have refined our monitoring capacities, complemented by integration with MRI and microscopy for richer cellular insights. Future directions point to machine learning for data interpretation, promising to unlock a deeper understanding of cellular thermodynamics.

## 6 Conclusion

Exploring cell temperature and thermogenesis underscores their complexity and pertinence in biology and medicine. This paper examines cellular heat generation mechanisms and innovative temperature measurement methods, split into 3 groups: localized contact, non‐contact, and global measurement methods. Each of them has their pros and cons. Contact methods affect the local cell temperature and can also damage the cells; non‐contact methods are not as precise as the contact ones, and the global methods are the most accurate, but they do not provide local temperature, only a global one. All methods combined offer new insights into addressing metabolic conditions and cancer therapies. Anticipated technological progress, including nanotechnology and advanced measurement methods, is set to expand our comprehension of cellular thermodynamics, offering promising avenues in both research and clinical innovation.

## Conflict of Interest

The authors declare no conflict of interest.
